# Mean Recency Period for Estimation of HIV-1 Incidence with the BED-Capture EIA and Bio-Rad Avidity in Persons Diagnosed in the United States with Subtype B

**DOI:** 10.1371/journal.pone.0152327

**Published:** 2016-04-11

**Authors:** Debra L. Hanson, Ruiguang Song, Silvina Masciotra, Angela Hernandez, Trudy L. Dobbs, Bharat S. Parekh, S. Michele Owen, Timothy A. Green

**Affiliations:** 1 Quantitative Sciences and Data Management Branch, Division of HIV/AIDS Prevention, National Center for HIV/AIDS, Viral Hepatitis, STD, and TB Prevention, Centers for Disease Control and Prevention, Atlanta, Georgia, United States of America; 2 Laboratory Branch, Division of HIV/AIDS Prevention, National Center for HIV/AIDS, Viral Hepatitis, STD, and TB Prevention, Centers for Disease Control and Prevention, Atlanta, Georgia, United States of America; 3 HIV Incidence and Case Surveillance Branch, Division of HIV/AIDS Prevention, National Center for HIV/AIDS, Viral Hepatitis, STD, and TB Prevention, Centers for Disease Control and Prevention, Atlanta, Georgia, United States of America; 4 International Laboratory Branch, Division of Global HIV and AIDS, Center for Global Health, Centers for Disease Control and Prevention, Atlanta, Georgia, United States of America; The University of Tokyo, JAPAN

## Abstract

HIV incidence estimates are used to monitor HIV-1 infection in the United States. Use of laboratory biomarkers that distinguish recent from longstanding infection to quantify HIV incidence rely on having accurate knowledge of the average time that individuals spend in a transient state of recent infection between seroconversion and reaching a specified biomarker cutoff value. This paper describes five estimation procedures from two general statistical approaches, a survival time approach and an approach that fits binomial models of the probability of being classified as recently infected, as a function of time since seroconversion. We compare these procedures for estimating the mean duration of recent infection (MDRI) for two biomarkers used by the U.S. National HIV Surveillance System for determination of HIV incidence, the Aware BED EIA HIV-1 incidence test (BED) and the avidity-based, modified Bio-Rad HIV-1/HIV-2 plus O ELISA (BRAI) assay. Collectively, 953 specimens from 220 HIV-1 subtype B seroconverters, taken from 5 cohorts, were tested with a biomarker assay. Estimates of MDRI using the non-parametric survival approach were 198.4 days (SD 13.0) for BED and 239.6 days (SD 13.9) for BRAI using cutoff values of 0.8 normalized optical density and 30%, respectively. The probability of remaining in the recent state as a function of time since seroconversion, based upon this revised statistical approach, can be applied in the calculation of annual incidence in the United States.

## Introduction

Measurement of the number of new HIV-1 infections per year and the annual rate at which incident infections occur is important for tracking HIV and monitoring transmission, for evaluation of preventive interventions, and for resource allocation. Application of laboratory methods to distinguish recent from non-recent HIV infection, based upon characteristics of the antibody response early after seroconversion, has led to a less costly approach in the estimation of HIV incidence [1]. Since the publication of a sensitive/less-sensitive testing algorithm in 1998 [2], a number of bioassays have been developed to detect recent infections [3–6]. Many of these assays have recently received a formal evaluation of performance as an incidence assay by the Consortium for the Evaluation and Performance of HIV Incidence Assays [7].

For future biomarker-based HIV incidence estimates using the stratified extrapolation approach [8], the U.S. National HIV Surveillance System (NHSS) will transition from using the Aware BED EIA HIV-1 Incidence test (BED) to the Bio-Rad avidity index (BRAI), a modification of the HIV-1/HIV-2 plus O ELISA test, to classify infections as recent vs. non-recent. The accuracy of new incidence estimates will depend on having an accurate estimate of the mean duration of recent infection (MDRI) and an accurate estimate of the distribution of recency duration from a representative sample of U.S. subtype B incident HIV infections. It is important to estimate MDRI for both BED and BRAI using the same statistical procedures to facilitate bridging between NHSS historic and future trends in HIV incidence.

A critical parameter in the estimation of HIV incidence, MDRI for a given bioassay such as BED or BRAI, is defined as the average length of time, over a fixed time T, that persons with newly acquired infection are classified by the bioassay as having recently acquired infection. MDRI is expressed as $\;\Omega_{T}\;=\;\int \begin{array}{l} T\\ 0 \end{array}\;P_{R}\left(t\right)dt$, where *P*_*R*_(*t*) is the probability of obtaining a recent result and t is the time since detectable infection. T was chosen to capture most of the dynamic range of a bioassay; i.e., P_R_ ≈ 0 for t>T [9]. Estimation of Ω_*T*_ consists of analyzing longitudinal data from HIV seroconverters, where standard methods, such as survival analysis or binomial regression, must be carefully adapted to consider fluctuations of HIV-infected persons in and out of the bioassay-defined state of recent infection and imprecisely known seroconversion times. This report details two general approaches used to estimate Ω_T_ and provides estimates of the Ω_*T*_ incidence parameter for both BED and BRAI for persons infected with subtype B, the predominant subtype in the United States. We also describe the distribution of recency duration for application to NHSS HIV incidence data.

## Materials and Methods

Biomarker data were generated from longitudinal specimens of therapy-naïve individuals from the United States infected with HIV-1 subtype B who had known dates of last negative and first positive HIV antibody test dates less than 365 days apart collected as part of various cohort studies, including: plasma samples from the HIVNET HIV network for prevention trials [10], the AIDSVAX B/B candidate vaccine phase III trial (Vax004) [11], the multicenter AIDS cohort study (MACS) [12], and the Seroconversion Incident Panel Project (SIPP) in collaboration with SeraCare Life Sciences, Inc. in Milford, MA. In addition, biomarker data were generated from commercially available seroconversion panels obtained from SeraCare Life Sciences (previously known as Boston Biomedical Inc. (BBI)) (Table 1). Specimens were unlinked from personal identifiers and determined not to be human subjects research by the Centers for Disease Control and Prevention.

**Table 1:** Characteristics of data used in estimation of MDRI by data source. The median and interquartile range of distributions are given for the HIV-negative seroconversion interval, i.e., time between last HIV-negative and first HIV-positive tests; the HIV-positive follow-up, reflecting the total duration of observation after testing positive; the number of HIV-positive samples; and the HIV-positive sampling intervals or times between consecutive samples.

Data Source (# subjects, # observations)	Bioassays tested by data source	HIV-Negative Seroconversion Interval (Days)	HIV-Positive Follow-up (Days)	# HIV-Positive Samples	HIV-Positive Sampling Intervals (Days)
**BBI (17, 50)**	BED	7 (5, 18)	7 (0, 21)	2 (1, 4)	5 (4, 11)
HIVNET (89, 380)	BED, BRAI	183 (148, 195)	217 (64, 517)	4 (2, 6)	77 (50, 91)
MACS (41, 154)	BED	182 (155, 192)	581 (456, 732)	4 (4, 4)	188 (161, 203)
SIPP (11, 95)	BRAI	30 (21, 56)	336 (184, 605)	9 (7, 11)	30 (28, 34)
Vax004 (62, 274)	BED, BRAI	184 (161, 196)	124 (65, 167)	4 (4, 5)	28 (22, 23)
All Sources (220, 953)	-	180 (114, 192)	208 (65, 510)	4 (3, 5)	51 (28, 95)

The principles of the BED-capture enzyme immunoassay, measuring the proportion of immunoglobulin G that is specific to HIV, and the Bio-Rad GS HIV-1/HIV-2 Plus O EIA, measuring the avidity of maturing antibodies to bind less strongly to the antigen early after infection, have been described in detail elsewhere [4, 13–14]. The thresholds used to classify recent vs. long-standing infection were 0.8 normalized optical density (OD) for BED and 30% for BRAI [15–16].

Two general approaches were used to estimate Ω_T_: (i) survival time methods, and (ii) binomial models of the probability of testing recent as a function of time since seroconversion. The fitted models were used to calculate Ω_T_ at the specified recency thresholds and within the dynamic range of each bioassay, at T = 2 years post seroconversion. Differences in survival time distributions by cohort were assessed using a log-rank test with a p-value adjustment for multiple comparisons.

In the first approach, the Kaplan-Meier estimator of the survival function describing the probability of being in the recent state as a function of time since seroconversion was estimated by a step function using a maximum likelihood approach, after approximating entry (seroconversion) and exit (transition from recent to non-recent) times for each subject. To use the Kaplan-Meier estimator, the time spent in the recent state must be known or right- censored (have a lower bound). Before application of the Kaplan-Meier estimator, seroconversion times were approximated by the midpoints of seroconversion intervals, i.e., the time between last HIV-negative and first HIV-positive tests. This approximation assumes the seroconversion times were uniformly distributed within the seroconversion intervals. The times of transitions from recent to non-recent infection were estimated using linear interpolation or regression (see below) between measurement readings. If a person did not have a non-recent result, then the time in the recent state was right-censored; i.e., time in the state was greater than the time from estimated seroconversion to the subject’s last visit (lower bound for survival time). Given the resulting set of distinct times spent in the recent state after seroconversion, the estimator for the probability of being in the recent state at time t post-seroconversion is given by $\hat{\text{S}}\left(\text{t}\right)\;=\;\prod_{t_{i}<\text{t}}\frac{n_{i}\;-\;d_{i}}{n_{i}}$, where d_i_ is the number of transitions from recent to non-recent occurring at time t_i_ post-seroconversion, and n_i_ is the number of subjects in the sample at time t_i_ after seroconversion (the number of subjects who have not yet transitioned and not been censored by time t_i_). $\hat{S}\left(t\right)\;=\;1$ for t<t_1_. Ω_*T*_ was estimated by $\int \begin{array}{l} T\\ 0 \end{array}\;\text{S}\left(\text{t}\right)dt$. The survival probability function was computed from the Kaplan-Meier step function, with probabilities given at each day between 0 and T days. Gaps between days were filled with the probability for the last day before the gap, and for those days with an estimated probability that included a fraction of a day, a weighted sum of probabilities was computed for the day, with weights based upon the duration per fraction. A correction to the Kaplan-Meier estimator was applied by classification of the maximum observation to an event if it was censored [17]. In addition to non-parametric survival, survival times spent in the recent state, as defined above, were modelled parametrically by the Weibull distribution. The probability distribution function for the Weibull distribution is given by $\text{g}\left(\text{t},\;\alpha ,\;\beta \right)\;=\;\frac{\alpha }{\beta }{\left(\frac{t}{\beta }\right)}^{\alpha -1}\text{exp}({\left(-\frac{t}{\beta }\right)}^{\alpha })$, where β is the Weibull scale parameter or spread of the distribution and α shape parameter.

Neglected in our previous estimates of MDRI [18], subjects observed often enough during the phase when the bioassay was near the recent/non-recent threshold may exhibit multiple fluctuations back and forth between the two states. For these subjects, predicted exit times were computed from linear regression of the bioassay measurement values on approximated days since seroconversion using all data between the observation prior to the time when the threshold was first reached and either the first observation above after the last value below the threshold or the last observation for those individuals not observed above the threshold at the end of their follow-up. If the prediction slope was negative, time was right-censored at the observation prior to when the threshold was first reached.

In the second adapted approach [19], the probability of testing recent was modeled by fitting a binary value for the observed non-recent/recent classifications, instead of the original OD or avidity value for BED and BRAI, respectively. A linear binomial regression model, assuming a logit or inverse sigmoidal logistic parametric form for the probability of testing recent as a function of time since seroconversion, i.e. g(p) = log(p/(1 − p)), was fit to the data using a maximum likelihood approach. Model goodness of fit was assessed using the AIC criterion [20]. The model form was g(P_R_(t)) = Σβt, where g(.) was the logit link function and Σβt the linear predictor. The seroconversion times were approximated by the midpoints of the seroconversion intervals prior to model fitting. Ω_*T*_ was estimated by $\int \begin{array}{l} \text{T}\\ 0 \end{array}{\text{g}}^{-1}(\sum \hat{\beta }\text{t})\text{dt}$ using the trapezoidal rule for 731 days (0 to T). The predictor was log-transformed time since seroconversion. Bootstrapping was performed by sampling from subjects; 1000 replicates were computed. To account for potential subject level clustering effect on estimates of MDRI, a random intercept binomial model, with the logit parametric form was fit to the data using a pseudo-likelihood technique. [21] The linear predictor is of the form Σβt + γ, where γ is the subject-specific random effect. The minimum and maximum cluster size per subjects was 1 and 15, respectively.

In addition to this parametric approach, a generalized additive model (GAM) was fit to the binomial data with a logit link. The additive model generalizes the linear binomial model by modeling the expectation of testing recent as g(P_R_(t)) = ΣΨ(*t*), where ΣΨ(*t*) was a smooth function of time since seroconversion, estimated non-parametrically with flexible spline terms for the predictor [22]. The degrees of freedom determine the amount of smoothing. A cross-validation method was used to optimize the effective number of parameters defined by the degrees of freedom [23]. The ‘log-transformed time since seroconversion’ term was fit by using a univariate smoothing spline with four degrees of freedom; of these, one was taken up by the linear portion of the fit and three by the nonlinear spline portion. As with the parametric binomial model, bootstrapping was performed to derive measures of accuracy.

SAS software, version 9.3 (SAS Institute Inc., Cary, NC, USA) was used to implement all steps of the estimation methods.

## Results

Collectively, 953 specimens from 220 HIV-1 subtype B seroconverters taken from 5 cohorts were tested using either BED, BRAI, or both (Table 1, S1 and S2 Tables). The median time between last HIV-negative and first HIV-positive specimens, based upon Western blot diagnostic testing criteria, was 180 (interquartile range (IQR) 114, 192) days. The median duration of follow-up following HIV-positive diagnosis was 208 (IQR 65, 510) days; there were a median of 4 (IQR 3, 5) specimens per subject sampled approximately every 51 (IQR 28, 95) days. The percentage of subjects who did not enter the non-recent state during the course of their follow-up, as defined by the bioassay threshold, was 29% (61/209) for BED and 38% (62/162) for BRAI. There were not significant differences in the BED or BRAI probability distributions for remaining in the recent state by cohort (p>0.05).

**Fig 1:**
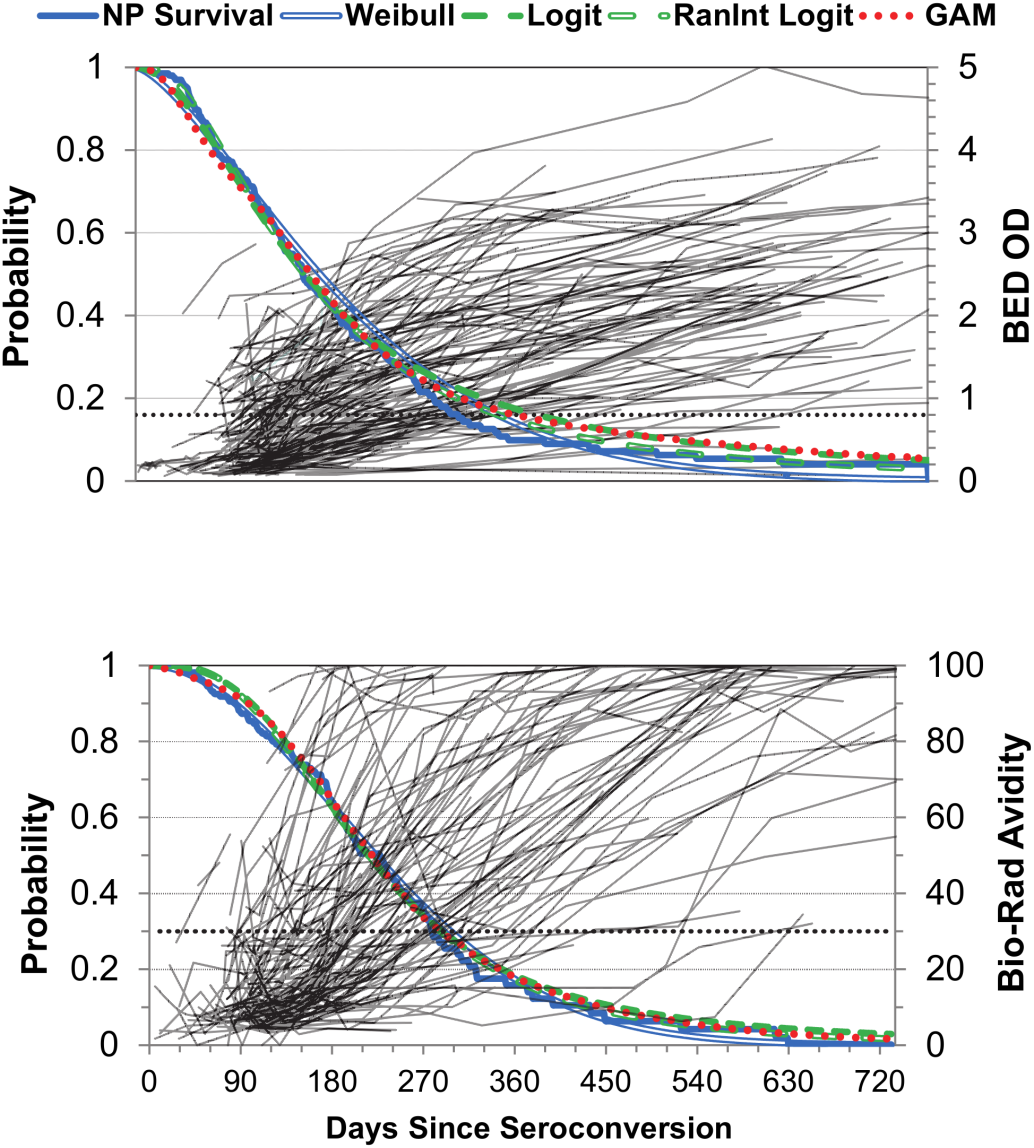
The probability of remaining in the recent state as a function of time since seroconversion for BED and BRAI bioassays. Model predicted probabilities from five estimation methods are given; blue lines represent the survival methods, green dashed lines the binomial logit, and red dotted line the GAM method. Subject-specific increasing trends in normalized OD or avidity are graphed in the background on the secondary vertical axis. The horizontal dashed line represents the bioassay threshold for classification of recency/non-recency (BED threshold 0.8; BRAI threshold 30%).

Most subjects show increases in HIV antibody levels over time, though there was heterogeneity among individual responses (Fig 1) for both BED and BRAI. The estimates for Ω_*T*_ range from 198.4 to 215.7 days for BED and 239.3 to 253.6 for BRAI avidity, differences of approximately 2–3 weeks between estimation methods (Table 2). The relative standard error (RSE), i.e. standard error divided by the mean, ranged from 5.8 to 8.0% for BED and 5.4 to 7.0% for BRAI, with the lower RSEs associated with the survival procedures or the binomial random intercept models, suggestive of slightly better fit, but with similar dispersion observed for the two bioassays.

**Table 2:** Estimated days of mean duration of recent infection (MDRI) and standard error, in parentheses, for BED and BRAI incidence bioassays. A total of 858 measurements from 209 subjects of 4 cohorts and 749 measurements from 162 subjects of 3 cohorts were used to estimate BED and BRAI MDRI, respectively. Results for five estimation procedures are given.

MDRI Estimation Method	BED, Cutoff 0.8	BRAI, Cutoff 30%
Non-Parametric Survival	198.4 (13.0)	239.6 (13.9)
Parametric Weibull Survival	198.8 (11.6)	239.3 (12.8)
Parametric Binomial Logit	215.7 (15.6)	253.6 (16.2)
Parametric Binomial Logit with Random Intercept	204.0 (13.1)	246.5 (13.2)
Non-Parametric GAM	213.8 (17.1)	249.7 (17.5)

## Discussion

In this report we have used five estimation procedures based on two statistical approaches to estimate the MDRI, a key parameter in estimation of population level HIV incidence, and the distribution of time from seroconversion to non-recent classification which can be used by NHSS to estimate the number of new HIV-1 infections for monitoring of trends in incidence at the national and state levels. Resulting estimates of Ω_*T*_ ranged from 198.4 to 215.7 days for BED and 239.3 to 253.6 days for Bio-Rad avidity. These results constitute revised estimates for the BED mean duration of recency used in the HIV-1 incidence algorithm [18] and are consistent with other recently published estimates of BED MDRI [19, 24]. Differences in Ω_*T*_ due to revisions will be inversely proportional to differences in estimated incidence when Ω_*T*_ is applied to NHSS incidence data because this parameter is in the denominator of the incidence algorithm [20, 25–26], and will result in lower estimates of HIV-1 incidence than previously published [27, 28]. Improved estimation methods that account for the wide variability in individual response to antibody maturation, and specifically the potential fluctuations around the bioassay threshold, have been implemented. In addition, we have developed a random intercept model for the binomial approach to appropriately account for varying subject-level cluster sizes. The resulting MDRIs based upon our data were 1–2 weeks shorter when estimated from the random effects models relative to the logit models which assumed independence of observations.

Estimation of Ω_*T*_ in general, and our estimation work in particular, have important limitations. Though we restricted our analyses to study cohorts and commercially available seroconverter panels of data from the United States for estimation of Ω_*T*_, a key difference from previously published results, these data may not be representative of all newly diagnosed HIV-1 subtype B infections in the United States. In addition, new diagnostic testing recommendations may lengthen MDRI by shortening the duration of time between transmission and detected infection. Future estimates will require an adjustment to MDRI or use of new seroconversion panel data from subjects diagnosed consistent with new HIV diagnostic testing algorithms. An important assumption is that of uniformly distributed seroconversion times in the seroconversion interval, the time from last HIV-negative to first HIV-positive tests. The assumption of uniformly distributed seroconversion times may be reasonable for individuals enrolled in studies that schedule visits. However, it may otherwise be possible to observe lack of independence between testing and seroconversion time for persons motivated by suspicion of infection or with concomitant needs for clinical care, e.g., diagnoses of STDs or pregnancy [29–32].

Results from simulation studies demonstrated that the estimation methods described in this report were robust to most data issues, e.g., duration of time between last HIV-negative and first HIV-positive tests, frequency of HIV-positive sampling, and loss-to-follow-up [33]. However, when data issues were compounded, e.g., when both HIV-negative and HIV-positive sampling frequency was lengthy, absolute bias increased. Although a number of methods can be used to estimate Ω_*T*_, there were differences in accuracy and precision depending upon the data quality. For example, models we implemented in estimation of MDRI that relied on a parametric assumption or ignored within-subject correlation in the presence of varying cluster sizes (measurements per subject) did not perform as well as the non-parametric survival approach in the presence of increasing measurement noise with T = 2 years. Although the estimates reported herein will require validation from other studies of seroconverters, the MDRI estimates based upon the revised survival approach, 198.4 days for BED and 239.6 days for BRAI, and more specifically, the probability of remaining in the recent state as a function of time since seroconversion, will be applied in calculation of annual incidence in the United States as described previously [25, 27].

## Supporting Information

S1 Table Spreadsheet with BED OD values at times since estimated time of seroconversion, assayed for 858 specimens from 209 HIV-1 subtype B seroconverters, taken from 4 data cohorts. (XLSX)

S2 Table Spreadsheet with Bio-Rad avidity values at times since estimated time of seroconversion, assayed for 749 specimens from 162 HIV-1 subtype B seroconverters, taken from 3 data cohorts. (XLSX)
